# Best evidence toolkits: a case study on interventions for preventing violence against women and girls (VAWG)

**DOI:** 10.1186/s13643-025-02798-z

**Published:** 2025-05-16

**Authors:** Michelle Richardson, Theo Lorenc, Katy Sutcliffe, Amanda Sowden, James Thomas

**Affiliations:** 1 EPPI Centre, UCL Social Research Institute, University College London, London, UK; 2 Centre for Reviews & Dissemination, University of York, York, UK

**Keywords:** Toolkits, Best evidence, Knowledge translation, Overviews

## Abstract

Research teams report challenges in conducting overviews and many of these relate to the synthesis of outcome data from multiple reviews that lead to unclear evidence. This limits research from being used by policymakers and other review users who need accessible robust evidence. In this commentary, we present a case study on creating a toolkit of interventions for preventing violence against women and girls (VAWG). This toolkit is underpinned by systematic methods and a priori criteria that identify a single best up-to-date systematic review of each subtopic. The best evidence toolkit approach does not require the synthesis of multiple reviews and produces clear, standardised evidence across subtopics efficiently. This approach offers a pragmatic alternative to overviews when presenting a broad spectrum of intervention approaches, populations or outcomes. This approach may be particularly beneficial when the primary aim is to communicate with policymakers.

## Background

### Overviews and the need for broad evidence synthesis

Policymakers and other research users need easy access to user-friendly and high-quality evidence. However, the sheer volume of evidence produced can make this a challenge. For instance, the increase in systematic reviews—involving the synthesis of primary studies—has led to a need for synthesising existing systematic reviews, resulting in overviews of systematic reviews (or ‘overviews’ for short) [8]. Whilst this synthesis can be beneficial it can also lead to conflicting findings when there are multiple reviews on a topic, making it difficult for decision-makers to draw clear conclusions [3, 5, 8]. For example, review authors can interpret findings differently due to variations in inclusion criteria or summary methods, while the over-representation of single studies across multiple reviews can lead to duplication bias. Despite methodological advancements [8], research teams continue to report challenges in synthesising findings across reviews. Given these complexities, overviews are often resource-intensive and require significant expertise [5].

### Evidenced based toolkits

Toolkits usually offer a package of information, resources, or tools that together support users to implement evidence-based recommendations into practice [1, 2]. While there is no commonly agreed definition they typically are rooted in the principle of evidence-based practice and involve the use of current best evidence to inform decisions. They have been designed for both single interventions [11] and broader approaches that consider a range of different interventions [4, 12]. Modern toolkits are often digitised and have interactive elements that enhance accessibility, like overviews, some have become living resources that are continuously updated and refined [4, 12].

There is little guidance on the optimal methodological approach for producing a toolkit [6]. A scoping review found that 37% of health-based toolkits were underpinned by a literature scan or review (*n* = 31) [2], though it is unclear whether primary and/or review-level evidence was searched and how the information informed the creation of the toolkits. It has been concluded that there is a lack of transparency concerning the evidence underpinning toolkits and that they are rarely evaluated in terms of their knowledge translation [2].

The Youth Endowment Fund (YEF) toolkit is exemplary in its approach to summarising reviews from a diverse range of interventions aimed at preventing serious youth violence [12]. However, unlike ‘standard’ overviews that synthesise across multiple systematic reviews, the YEF’s methodology involves applying a priori criteria to select one best review to estimate the impact for each of a range of interventions. This ‘best evidence approach’ originally utilised in reviews of primary studies [10] emphasises the practical significance and generalisability of findings using the most relevant and highest quality studies (here reviews) to inform practice and policy. Similar to ‘standard’ overviews, quality assessment is conducted at the review level to help identify potential biases in the review process. In addition to evidence of impact, the YEF toolkit offers insights into costs and implementation, with the latter drawing on qualitative review data and supplemental searches where needed.

Drawing on the YEF toolkit, we adopted a best-evidence approach to build a toolkit based on an overview of intervention approaches for preventing violence against women and girls (VAWG) in healthcare settings.

### Best evidence toolkit case study on interventions for preventing violence against women and girls (VAWG)

The starting point for the VAWG toolkit (EPPI-Vis (ioe.ac.uk))^1^ was a broad database search for evidence on VAWG, using a filter for systematic reviews^2^ and a fairly strict date limit (10 years) (further detail on the methods can be found in the methods guide by Lorenc et al. [7]). After screening the results for topic and method, we sorted them a posteriori into broad intervention and population categories (e.g. psychological therapies for victims of intimate partner violence); in total there were 18 categories. We then applied the AMSTAR-2 (A Measurement Tool To Assess Systematic Reviews 2) [9] to all the reviews and derived an overall evidence quality score for each (‘high’, ‘moderate’, ‘low’ or ‘critically low’). We then selected the ‘best’ available review within each category, i.e. one which (a) reported a meta-analysis of effectiveness data (where available), (b) had the highest AMSTAR-2 score, and (c) in case of a tie, the most recent search date.

We then prepared a summary for the front page of the toolkit’s digital interface, categorising the following dimensions of the effectiveness reviews:

- The effect size (categorised as ‘none’, ‘small’, ‘moderate’ or ‘large’ impact based on the size of the standardised mean difference and statistical significance of the effect);
- The evidence quality (using the AMSTAR-2 summary ratings);
- The review size (the number of primary studies included in relevant meta-analyses used to derive effect sizes); and
- The cost of the intervention (categorised as ‘low’, ‘moderate’ or ‘high’ based on supplementary searches for cost data, calculated per participant)

**Fig. 1:**
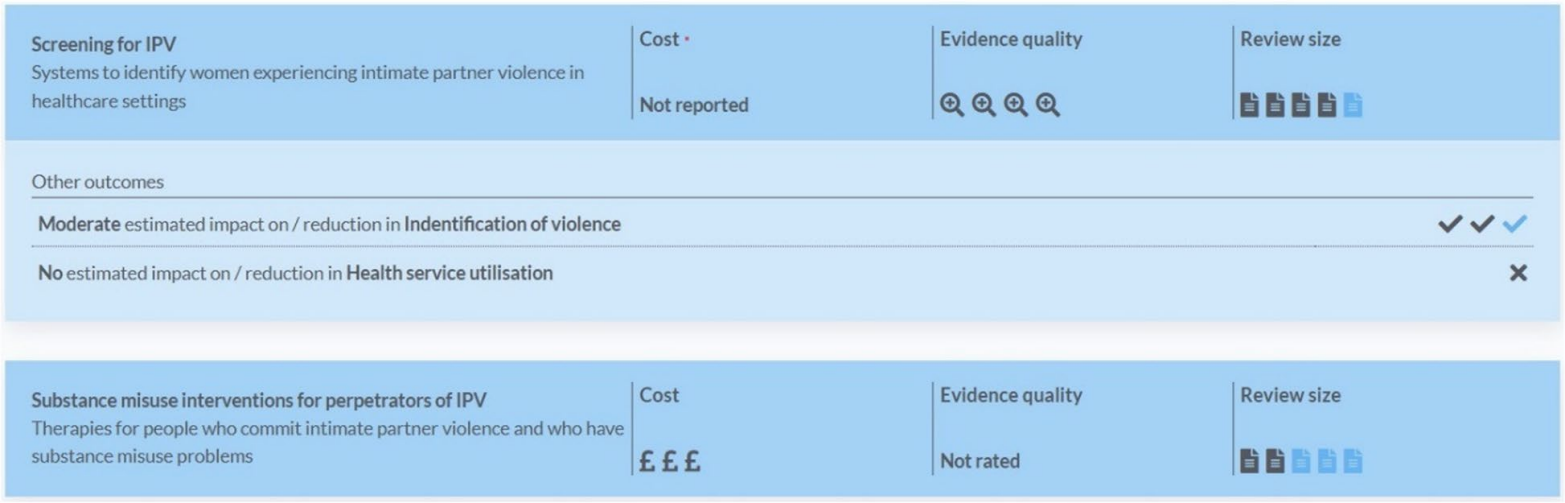
Example of summaries on the front page of the toolkit

An example of these summaries can be found in Fig. 1. Each summary on the front page of the toolkit links first to a brief description of the intervention and the effectiveness evidence for that topic, along with information on costs and implementation derived from supplementary searches, and then to a technical report giving full details.

## Conclusions

The creation of evidence toolkits may offer a pragmatic and efficient approach for bringing together and presenting large, diverse bodies of evidence—particularly those which cover a heterogeneous range of populations, interventions and/or outcomes—which can arguably avoid some of the challenges of overviews. The key point is that the ‘best evidence’ approach adopted here does not require synthesis of review-level evidence; rather, it aims to identify a single high-quality and up-to-date review for each topic and then to summarise evidence across topics in a standardised and easily accessible format.

This is almost certainly more resource-efficient than producing an overview, since it only requires broad topic coding and quality assessment of all available reviews, with no further data extracted from those reviews not selected for the summary. By extension, it will probably be easier to update and maintain than a conventional overview. (This said, preparing a user interface like the one created for this toolkit requires resources and specialist skills). Best evidence toolkits may avoid some of the methodological pitfalls of overviews, such as double-counting of primary studies. Perhaps the main argument in their favour, though, is that they can direct decision-makers and other research users to the best available evidence in an accessible and concise way, without the inevitable lack of clarity which comes from synthesising diverse and partially overlapping reviews. Anecdotally, our conversations with policy colleagues suggest that evidence toolkits are valued for their accessibility and ease of use, although further research is required to understand how users access toolkits and the information presented.

Inevitably, there are some unresolved questions and challenges. The standardisation of messages across topics involves a large degree of ‘flattening out’ which may be misleading. For example, our toolkit covered a wide range of outcomes, including both health status outcomes (e.g. incidence of violence, or mental health outcomes for victims) and intermediate outcomes (e.g. knowledge or attitudes) which are often not clearly connected to the ultimate goals of interventions. While this is a problem for overviews too, the ease of drawing comparisons across topics in the toolkit format makes it potentially a more serious challenge in terms of users’ understanding of the evidence. (An alternative approach—taken by the YEF’s toolkit, for example—is to ‘translate’ diverse outcomes into a single metric, but this has other challenges of its own).

In conclusion, we suggest that the evidence toolkit approach may be worth considering as an alternative to overviews of effectiveness reviews, especially where accessible communication of results to decision-makers or other stakeholders is the ultimate aim. By avoiding the attempt to comprehensively synthesise the findings of reviews and focusing on locating the best available evidence for each subtopic within a broadly defined field and standardising the presentation of data, toolkits can rapidly direct research users to the best available data to inform decisions. This commentary offers some suggestions as to the presentation of data, but of course, there are other options; each of which could be a focus of future research, as well as more user-centred questions about how audiences interact with and understand evidence toolkits.

## Data Availability

The data that support the findings of this study are available on request from the corresponding author.

## Abbreviations

VAWG: Violence against women and girls
YEF: Youth Endowment Fund
AMSTAR-2: A Measurement Tool to Assess Systematic Reviews 2

## Footnotes

1 Violence against women and girls toolkit (IOE.ac.uk); EPPI-Vis (ioe.ac.uk.). Accessed 2024–08-05.

2 Reviews, Medline–Maximises Specificity. In: McMaster Clinical Hedges database. Ontario: McMaster Health Knowledge Refinery; 2023: https://hiruweb.mcmaster.ca/hkr/hedges/medline/. Accessed 2023–04-03.
